# NGO online disclosures index in the presence of auxiliary information

**DOI:** 10.1371/journal.pone.0238297

**Published:** 2020-09-15

**Authors:** Ayesha Nazuk, Sadia Nadir, Ali R. Ansari, Raheel Nawaz

**Affiliations:** 1 School of Social Sciences and Humanities (S3H), National University of Sciences and Technology (NUST), Islamabad, Pakistan; 2 Department of Mathematics and Statistics, Faculty of Engineering and Applied Sciences, Riphah International University, Islamabad, Pakistan; 3 Department of Mathematics & Natural Sciences, Centre for Applied Mathematics & Bioinformatics, Gulf University for Science & Technology, Kuwait City, Kuwait; 4 Department of Operations, Technology, Events and Hospitality Management, Manchester Metropolitan University, Manchester, United Kingdom; Shandong University of Science and Technology, CHINA

## Abstract

This study highlights the need for analysis of online disclosure practices followed by non-governmental organizations; furthermore, it justifies the crucial role of potential correlates of online disclosure practices followed by non-governmental organizations. We propose a novel index for analyzing the extent of online disclosure of non-governmental organizations (NGO). Using the information stored in an auxiliary variable, we propose a new estimator for gauging the average value of the proposed index. Our approach relies on the use of two factors: imperfect ranked-set sampling procedure to link the auxiliary variable with the study variable, and an NGO disclosure index under simple random sampling that uses information only about the study variable. Relative efficiency of the proposed index is compared with the conventional estimator for the population average under the imperfect ranked-set sampling scheme. Mathematical conditions required for retaining the efficiency of the proposed index, in comparison to the imperfect ranked set sampling estimator, are derived. Numerical scrutiny of the relative efficiency, in response to the input variables, indicates; if the variance of the NGO disclosure index is less than the variance of the estimator under imperfect ranked set sampling, then the proposed index is universally efficient compared to the estimator under imperfect ranked set sampling. If the condition on variances is unmet, even then the proposed estimator remains efficient if majority of the NGO share online data on the auxiliary variable. This work can facilitate nonprofit regulation in the countries where most of the non-governmental organizations maintain their websites.

## I. Introduction

Over the past two decades, substantial developments in communication and collaboration technologies have transformed the world into a digitally interlinked space. Most organizations use multiple digital platforms to communicate with internal and external stakeholders. However, the organizational website is often the primary source of information for external stakeholders. Taking a historic view, Friedman [1] discussed the ten “flatteners” that have transformed our world: Collapse of the Berlin Wall in 1989, introduction of Netscape in 1995, workflow software (e.g., ProWorkflow, Nintex, and Dapulse), uploading information on the internet, offshoring, outsourcing, supply-chaining, insourcing, informing through search engines (e.g., Bing, and Google), wireless communication devices (which were highlighted as the “steroids”), and file-sharing tools [1]. Although Friedman [1] did not allude towards the accountability practices that organizations should adhere to; a cogent argument can be inferred that in such an interlinked world, transparent norms of sharing information will imply better accountability trends among the organizations [1].

Kahneman [2] discussed the idea that human beings are not always rational agents. He theoretically delineated the human decision-making process into two systems: System-I that takes quick decisions in urgent scenarios, and system-II that is invoked for reflective and complex decision-making. Non-governmental organizations (NGO) can shape public opinion by impressing upon both systems of human cognition. NGO can selectively prioritize the mention of certain topics on their websites; thereby, influencing the public through availability bias, i.e., the tendency of human beings to consider available information more important than the absent information.

This backdrop mandates the need for better accountability practices among NGO. Given that NGO are usually nonprofit organizations, their efficiency should be defined in terms of their capacity, and ability to achieve social goals in the thematic area that they are serving [3]. While globalization has become a cliché, the neologism “Global Administrative Law (GAL)” is probably a more specific term [4]. GAL purports the idea of global governance. Proponents of this notion assert that the world is one global administrative space; therefore, international regulatory institutions should monitor the economic, political, and social dynamics of individual states. They argue for trans-governmental regulatory paradigms through international organizations, respecting the interdependence of key domains of security, economic and social development, intellectual property rights, and analysis and regulation of human inter-country migrations. In the words of Kingsbury [5] GAL is explained as follows;

*“The term ‘law’ in GAL means a ‘body of rules’*, *which in this case regulate international organizations*, *global hybrid public-private or genuinely private institutions exercising public functions*, *states and both transnational and domestic civil societies”* [5].

GAL encompasses governance and administrative issues of the entire world, interweaving aforementioned issues at national, international, transnational, and domestic levels. GAL is a cosmopolitan approach of governance that perceives the world as a global constituency; therefore, it advocates the idea of international accountability standards that organizations should follow [6]. While the cosmopolitan school of thought is yet to arrive at a consensus in terms of the optimum paradigms for the institutionalization of accountability mechanisms for such an interdependent version of the world, they emphasize the need to give weight to the representations of NGO in global administrative space [6]. The ideas purported by the cosmopolitans have not gone unheeded; examples of involvement of NGO in the world affairs are numerous, such as NGO representation in the platform for addressing the complaints regarding projects funded by the World Bank [6]. A more specific example is the World Bank Inspection Panel that analyzes the provision of the most basic needs of the world; it has representation in the Codex-Alimentarius Commission which supports food legislation of the world [6]. The commitment of the World Trade Organization (WTO) towards the importance of NGO is manifested in the article V.2 of the Marrakesh agreement, which states that NGO can participate directly in the WTO negotiations for the purpose of transparency and consultative deliberations [7]. The WTO Decision WT/L/162 states that the WTO agreements should be analyzed by the WTO Secretariat in consultation with NGO to maximize transparency [8].

Building on the idea of a global administrative space, researchers have analyzed similarities and dissimilarities in the accountability paradigms of different countries. Nazuk [9] conducted an inquiry into the accountability practices followed in different countries. By analyzing a web-survey of 19 countries (from all human-inhabited continents), they checked three traits that support the accountability paradigm of NGO; the traits were, existence of a regulatory authority for NGO, existence of an independent NGO watchdog, and provision of a search-tab on the website of the aforementioned regulatory authority. They concluded that the performance of NGO from Europe and North America was the best, while the NGO from Asia and Africa performed worst, indicating a positive correlation between income group and the three aforementioned traits [9]. Out of 19 countries considered by Nazuk [9], the best performing trait was the existence of an NGO regulatory body, followed by the existence of search-tab on the website of the NGO regulatory body. The worst performance trait was the existence of an NGO watchdog body. More specifically, 89.5% of the sampled countries had NGO regulatory bodies, 63.2% had search-tabs on the websites of their NGO regulatory bodies, while only 31.6% had an independent NGO watchdog [9]. In the absence of either an NGO watchdog or an NGO sector regulatory body, the importance of independent evaluation of NGO’ websites is even more pronounced.

Many researchers have highlighted the importance of a registered website of an NGO [3, 10–12]. An NGO is mainly accountable in three forms: upward accountability, downward accountability, and internal accountability. Downward accountability focuses on efficient flow of information towards the NGO’s beneficiaries; internal accountability focuses on efficient flow of information within an organization; and upward accountability focuses on meeting information demands of donors, the host/funding government, and the government of the country where the NGO is operating [13, 14]. With the help of its website, an NGO can share crucial data, meeting the demands for the various aspects of accountability [15–17]. Online dissemination of accountability related information can provide opportunities for mutual information exchange between stakeholders, e.g., via a typical tool like a public blog on the NGO’s website [18]. It has been previously shown that the public is interested in understanding the impact of the nonprofit sector; for instance, Voitkane & Jakusonoka [19] analyzed the voluntary disclosure of financial information on the websites of public benefit organizations in Latvia. Despite the fact that 47% of the respondents in their survey showed interest in retrieving online information through an organization’s website, only 22% of the organizations share financial data [19]. Realizing the importance of the internet as an online tool for dissemination of information, researchers have designed indices to monitor the quality of information shared online by the NGO. Boire and Prakash [20] designed a 7-dimensional accountability index that can be used to evaluate the online disclosure practices followed by the NGO working in the USA. The aforementioned dimensions are as follows: beneficiary responsibility (4 elements), codes and standards (6 elements), employment responsibility (5 elements), environmental responsibility (4 elements), financial responsibility towards donors (10 elements), public responsibility (6 elements), and supplier responsibility (4 elements). Do, Davey & Coy [21] analyzed the quality of information shared by organizations in South Korea, through the Local E-government Accountability (LEGA) index. They considered three dimensions to construct the LEGA index: quality of general disclosures, intensity of financial information, and quality of the website as an online tool for accountability.

Developed countries often have more stringent legislation to circumvent information asymmetries in the nonprofit sector. For example, the NGO in the USA, except for faith-based nonprofits, can only claim exemption from tax, if they submit IRS form 990 to the Internal Revenue Services (US Department of Treasury; Title 26, section 501(c) of the Internal Revenue Code, is applicable for NGO working in USA) [22]. Therefore, submitting the IRS form 990 to the Internal Revenue Services is a mandatory action for NGO working in USA. This ensures that NGO share crucial information, for instance, total liabilities, total assets, information on donations of more than $25,000─ in non-cash form, contributions of historical treasures or similar assets, list of all current and previous employees along with data of their annual salaries, members of the board of directors, and list of contractors. Stakeholders can obtain any NGO’s IRS form 990 directly from the Internal Revenue Services [23] or download it from charity watchdogs working in the USA, such as Guidestar and Pro Publica [24, 25]. Charity Services regulates the NGO working in New Zealand; its website includes an interactive clickable map through which the public can stay abreast of live statistics about the charities working in different particular areas of the country, users can search for a charity by its name, street address, and registration number [26]. All charities working in New Zealand are required to submit annual returns data, performance report, and financial data to Charity Services. Moreover, Charity Services conducts a holistic audit of the charities that are registered with it; the audit process encompasses all the organizational phases, for instance, the form ISA (NZ) 265 pertains to analyzing the internal communication efficiency of those entrusted with governance, and management of the charity; the form ISA (NZ) 710 pertains to comparative temporal audit of financial statements [27].

Auxiliary information, such as total revenue and number of branches of an NGO, is linked with their online disclosure practices. Researchers have empirically shown that larger NGO are more likely to disclose (on their websites) rich information encompassing various dimensions of accountability [6, 28, 29]. Having established that auxiliary information can play an important role in explaining the information culture of NGO in the cyberspace, we took inspiration from two groups of researchers: those who discussed estimators for population mean in the presence of auxiliary information [30–37], and those who made use of auxiliary information in ranked set sampling schemes [38–41].

This study highlights the importance of the organizational websites, as a tool for better accountability. Our approach capitalizes on the observation that many NGO share data about an auxiliary variable i.e., total revenue; therefore, incorporating the data of total revenue, a new index is proposed to analyze the online disclosure practices of non-governmental organizations. While several applications of imperfect ranked-set sampling scheme can be found in research literature, we present a novel application of this sampling scheme for monitoring the quality of information shared online by the non-governmental organizations.

## II. Materials and methods

We propose a new estimator for analyzing the online disclosure practices of non-governmental organizations; the proposed index makes use of the information stored in an auxiliary variable. For online disclosure scores, we follow the NGO disclosure index constructed by Nazuk & Shabbir [16], which comprises three dimensions of online accountability: usability, content, and communication. From here on we shall refer to the index proposed by Nazuk & Shabbir [16] as NDI. The first dimension of NDI is the website’s usability, implying that the website content should be user-friendly and purposefully clear, for instance, a clickable link that routes to specific sections of websites, such as a clickable link labeled, “International Linkages”. Nazuk & Shabbir [16] defined 11 elements of usability: home page length, click ability, external links to international organizations, external links to local organizations, search tab, availability of website in more than one languages, site map, and the four WCAG2 [42] criteria i.e., perceivability, understandability, operability, and robustness.

*“Good usability is when we use something almost or completely without noticing that we are using an interface to do the thing we want to do (e*.*g*., *boxes of fruit juice with those nice plastic lids)*. *If we do notice the interface*, *it might be to register the pleasure of using that interface*. *Poor usability is when we get frustrated and the method/interface seems to be a barrier*, *stopping us from making progress (e*.*g*., *a friend gives you a lift in to get out*, *almost always a problem to do quickly and effectively)”* [43].

The second dimension of NDI is the (website) content. Effective website content ensures that stakeholders who visit the website are actively engaged; therefore, NGO should ensure that the website has no irrelevant content and is regularly updated. If the website content is not efficiently curated, then there is an exponential decrease in the probability of a visitor using the website [44]. Nazuk & Shabbir [16] included 32 elements of content: information about the website developer(s), information about copyrights, address of NGO’s nationwide office, mission statement, NGO’s strategic plan and goals, NGO’s background information, an active link for donations, information about past projects spearheaded by the NGO, list of programs, information about opportunities for volunteering, data about jobs and online jobs' portal, office location shown on a geographic information system, FAQs (frequently asked questions), calendar of events, use of media to apprise the users about the NGO, measures used to evaluate its performance, audited financial statements, annual reports, privacy policy, members of BOD (board of directors), minutes of BOD meeting(s), contact directory listing the employees, method to apply for membership/services/programs, community updates, newsletter(s), procedure for submitting feedback, NGO’s registration number, statistical proofs of performance, NGO bylaws, information about the law under which it is registered, information about certification & awards, physical addresses of branches operating in Pakistan.

The third dimension of NDI is the website’s quality of communication, which has three elements: link to social networking sites, a blog, and profiling of surveys conducted for employees and beneficiaries.

Each element can be given a score from 0 to 3, depending upon the quality of disclosures, for instance, for the dimension of “content”, if no annual report is shared, then a zero score is awarded to the element “annual reports”; if only the latest annual report is shared (while the age of NGO is greater than one), then the score is 1.5; and if full archives of annual reports is available, then the score is 3.

This study offers significant improvement in the index proposed by Nazuk & Shabbir [16] by incorporating vital information from the NGO website. An additional advantage of the proposed methodology is the use of ranked set sampling that has better performance than the simple random sampling [38–41]; Nazuk & Shabbir [16] used simple random sampling. Suppose that [(100)(*ϖ*)]% NGO share online information on all the elements of the three dimensions of NDI, while [(100)(1−*ϖ*)]% NGO share information on these dimensions and total revenue, then we propose the following estimator for the mean score of online disclosure index for NGO; from hereon we shall refer it as NDIA (NGO Disclosure Index under Auxiliary Information; mathematically expressed as ${\hat{\mu }}_{NGO.Disclosures}$). It has been observed that some NGO share auxiliary information on their websites; such information may include total revenue. Imperfect ranked set sampling is used as we can rank the NGO according to the auxiliary information; literature indicates that imperfect ranked set sampling is more efficient than the simple random sampling [38–41]. By doing so, we are ranking the NGO with minimal calculations; we only need to note that value of the auxiliary variable.

$${\hat{\mu }}_{NGO.Disclosures}=\varpi {\hat{\mu }}_{NDI}+\left(1-\varpi \right){\hat{\mu }}_{IRSS},$$ (1)

$$\mathrm{where}\;\begin{array}{l} {}[{\hat{\mu }}_{NDI}=w_{1}{\hat{\mu }}_{Usabilty}+w_{2}{\hat{\mu }}_{Content}\\ +(1-w_{1}-w_{2}){\hat{\mu }}_{Communication}] \end{array},$$ (2)

Nazuk and Shabbir [16] derived the optimum values of *w*_1_ and *w*_2_, the optimum values are given in Eq (3).

$$\begin{array}{l} W_{1}=\frac{1}{1+\frac{V(X_{U})\left[V\left(X_{C}\right)+V\left(X_{Com}\right)\right]}{V\left(X_{C}\right)V\left(X_{Com}\right)}},\\ V\left(X_{k}\right)=p_{k}q_{k};\;X_{k}∼Binomial\left(p_{k}\right),\;k=U,C,Com,\\ W_{2}=\frac{1}{1+\frac{V(X_{C})\left[V\left(X_{U}\right)+V\left(X_{Com}\right)\right]}{V\left(X_{U}\right)V\left(X_{Com}\right)}}. \end{array}]$$ (3)

Numerical values of these weights are dependent upon the probabilities of NGO meeting the threshold values of usability, content, and communication; these thresholds can be taken from a study conducted in a similar environment. For instance, Denmark and Finland follow restrictive regulatory style of NGO regulation, macro-institutions follow corporatism; therefore, if a study conducted in Finland finds that the usability score for NGO’ websites is 15, then we can use *p*_*U*_ = (*Usabilty Score*≥15), for Denmark, so, if 30% of the NGO in Denmark score at least 15 on usability then *p*_*U*_ = 0.3 [22]. Another approach proposed by Nazuk [9], is to use sample average of score on each dimension as an estimate of the thresholds i.e., if the average score of usability is 2, then we can use it as an estimate of the threshold for usability. The optimum variance of ${\hat{\mu }}_{NDI}$ is given by the equation;

$$\begin{array}{l} V{\left({\hat{\mu }}_{NDI}\right)}_{opt}={\left\{\frac{1}{1+\frac{V(X_{U})\left[V\left(X_{C}\right)+V\left(X_{Com}\right)\right]}{V\left(X_{C}\right)V\left(X_{Com}\right)}}\right\}}^{2}V(X_{U})+\\ +{\left\{\frac{1}{1+\frac{V(X_{C})\left[V\left(X_{U}\right)+V\left(X_{Com}\right)\right]}{V\left(X_{U}\right)V\left(X_{Com}\right)}}\right\}}^{2}V(X_{C})+.... \end{array}$$

$$....+{\left[1-\left\{\frac{1}{1+\frac{V(X_{U})\left[V\left(X_{C}\right)+V\left(X_{Com}\right)\right]}{V\left(X_{C}\right)V\left(X_{Com}\right)}}\right\}-\left\{\frac{1}{1+\frac{V(X_{C})\left[V\left(X_{U}\right)+V\left(X_{Com}\right)\right]}{V\left(X_{U}\right)V\left(X_{Com}\right)}}\right\}\right]}^{2}V\left(X_{Com}\right)]$$ (4)

Having discussed the procedure for calculation of ${\hat{\mu }}_{NDI}$, we now proceed to the procedure for computing ${\hat{\mu }}_{IRSS}$ i.e., the estimated average online disclosure score derived through the imperfect ranked set sampling scheme; using total revenue as the auxiliary variable. To calculate ${\hat{\mu }}_{IRSS}$ we propose the procedure explained below;

(a) Draw a random sample of *m* NGO; rank these NGO in accordance of their total revenues. (b) For the NGO with minimum value of total revenue, calculate the value of NDI by using Eqs (2) and (3).
Repeat (1) (a), for the NGO with the second minimum value of total revenue calculate the value of NDI by using Eqs (2) and (3).
Repeat step (1) and (2) *r* times to complete *r* cycles of the imperfect ranked set sampling scheme. This procedure generates a sample of size *n*, where *n* = *mr*.
Let X_*i*(*i*:*m*)[*j*]_ represents the value of NDI on the *i*^*th*^ measured NGO with rank *j* i.e., NGO with *j*^*th*^ smallest value of total revenue, *j* = 1,2,….*r*, and *r* represents the cycle number. An illustration of the 1^st^ cycle of this procedure is given in Table 1.

**Table 1:** Sample layout of 1^st^ cycle of imperfect ranked set sampling procedure.

Set 1	Set 2	Set 3	Set m
X_1(1:*m*)[1]_	X_1(2:*m*)[1]_	X_1(3:*m*)[1]_	X_1(*m*:7)[1]_
X_2(1:*m*)[1]_	X_2(2:*m*)[1]_	X_3(2:*m*)[1]_	X_*m*(*m*:*m*)[1]_
$\begin{array}{c} .\\ . \end{array}$	$\begin{array}{c} .\\ . \end{array}$	$\begin{array}{c} .\\ . \end{array}$	$\begin{array}{c} .\\ . \end{array}$
X_*m*(1:*m*)[1]_	X_*m*(2:*m*)[1]_	X_3(*m*:7)[1]_	X_*m*(*m*)[1]_

The variance expression for ${\hat{\mu }}_{IRSS}$ is given below.

$$\begin{array}{l} V\left({\hat{\mu }}_{IRSS}\right)=V\left(\frac{1}{mr}\sum_{i=1}^{m}.\sum_{j=1}^{r}Y_{i\left(i:m\right)\left[j]\right]}\right)=\frac{1}{{\left(mr\right)}^{2}}\sum_{j=1}^{r}.\sum_{i=1}^{m}V\left(\mu_{Y}+\frac{\rho_{XY}\sigma_{Y}}{\sigma_{X}}\left(X_{i\left(i:m\right)[j]}-\mu_{X}\right)+\varepsilon_{ij}\right)\\ V\left({\hat{\mu }}_{IRSS}\right)=\frac{1}{m^{2}r}\sum_{i=1}^{m}V_{X}E_{Y/X}\left[\left(\mu_{Y}+\frac{\rho_{XY}\sigma_{Y}}{\sigma_{X}}\left(X_{i\left(i:m\right)[j]}-\mu_{X}\right)+\varepsilon_{ij}\right)\right]\\ +\frac{1}{m^{2}r}\sum_{i=1}^{m}E_{X}V_{Y/X}\left[\left(\mu_{Y}+\frac{\rho_{XY}\sigma_{Y}}{\sigma_{X}}\left(X_{i\left(i:m\right)[j]}-\mu_{X}\right)+\varepsilon_{ij}\right)\right]\\ where\;\varepsilon_{ij}\;represents\;the\;random\;error\;induced\;due\;to\;the\;imperfect\;ranking\;scheme\;used\;to\;rank\;the\;NGOs. \end{array}]$$ (5)

$$V\left({\hat{\mu }}_{IRSS}\right)=\frac{\sigma_{Y}^{2}\left(1-\rho_{XY}^{2}\right)}{mr}+\frac{\rho_{XY}^{2}\sigma_{Y}^{2}}{\sigma_{X}^{2}}\frac{1}{m^{2}r}\sum_{i=1}^{m}\sigma_{X\left(i:m\right)}^{2},$$ (6)

where $\sigma_{Y}^{2}$ is variance of *Y* i.e., variation in the values of NDI, $\sigma_{X}^{2}$ is the variation in the values of *X* i.e., variation in the values of total revenue of NGO, $\rho_{XY}^{2}$ is the correlation between *Y* and *X*. We obtain the variance expression for ${\hat{\mu }}_{NGO.Disclosures}$ is given below;

$$V\left({\hat{\mu }}_{NGO.Disclosures}\right)=\varpi^{2}V\left({\hat{\mu }}_{NDI}\right)+{\left(1-\varpi \right)}^{2}V\left({\hat{\mu }}_{IRSS}\right).$$ (7)

To analyze the performance of ${\hat{\mu }}_{NGO.Disclosures}$ we compare it with ${\hat{\mu }}_{IRSS}$, so that we can comment whether the availability of data for total revenue, for [100*(1−*ϖ*)]% NGO improves the quality of estimation of the average NGO disclosure score in the population.

## III. Calculations

The mathematical condition for better performance of ${\hat{\mu }}_{NGO.Disclosures}$ as compared to ${\hat{\mu }}_{IRSS},$ is given below.

$$\begin{array}{l} \varpi^{2}V\left({\hat{\mu }}_{NDI}\right)+{\left(1-\varpi \right)}^{2}V\left({\hat{\mu }}_{IRSS}\right)<V\left({\hat{\mu }}_{IRSS}\right)\\ \varpi^{2}V\left({\hat{\mu }}_{NDI}\right)<V\left({\hat{\mu }}_{IRSS}\right)\left(1-1-\varpi^{2}+2\varpi \right)\\ \varpi^{2}V\left({\hat{\mu }}_{NDI}\right)<V\left({\hat{\mu }}_{IRSS}\right)\left(2\varpi -\varpi^{2}\right)\\ V\left({\hat{\mu }}_{NDI}\right)<V\left({\hat{\mu }}_{IRSS}\right)\left(\frac{2\varpi -\varpi^{2}}{\varpi^{2}}\right)\\ V\left({\hat{\mu }}_{NDI}\right)<V\left({\hat{\mu }}_{IRSS}\right)\left(\frac{2}{\varpi }-1\right)\\ \frac{V\left({\hat{\mu }}_{NDI}\right)}{V\left({\hat{\mu }}_{IRSS}\right)}+1<\frac{2}{\varpi } \end{array}]$$ (8)

$$\varpi <\frac{2}{\frac{V\left({\hat{\mu }}_{NDI}\right)}{V\left({\hat{\mu }}_{IRSS}\right)}+1}]$$ (9)

Note that if the denominator is less than 2, only then the condition on the weight is universally true; therefore, we reduce the above equation to the following form.

$$\begin{array}{l} \frac{V\left({\hat{\mu }}_{NDI}\right)}{V\left({\hat{\mu }}_{IRSS}\right)}+1<2\\ V\left({\hat{\mu }}_{NDI}\right)<V\left({\hat{\mu }}_{IRSS}\right) \end{array}]$$ (10)

The denominator of the term on the left-hand side of inequality (9), can be greater than 2; even then, the inequality may hold in some cases. For one such case, consider the equations that follow.

$$V\left({\hat{\mu }}_{IRSS}\right)=0.0036,\;V\left({\hat{\mu }}_{NDI}\right)=0.0833,\;\mathrm{then}\;\frac{2}{\frac{V\left({\hat{\mu }}_{NDI}\right)}{V\left({\hat{\mu }}_{IRSS}\right)}+1}<0.083\;or\;\varpi <0.083.$$

For the cases when the denominator is greater than 2, an analysis of the asymptotic behavior of the term on the right-hand side in inequality (9), leads to

$$\begin{array}{l} \frac{V\left({\hat{\mu }}_{NDI}\right)}{V\left({\hat{\mu }}_{IRSS}\right)}+1>2\\ V\left({\hat{\mu }}_{NDI}\right)>V\left({\hat{\mu }}_{IRSS}\right) \end{array}]$$ (11)

It must be noted that the inequality (11) is met only in some limiting cases; if the denominator is 4, then *ϖ* must be less than 0.5 to make the proposed estimator efficient. Note that the more the denominator is greater than 2, the more stringent the condition on *ϖ* is; for instance, if the denominator is 40, then *ϖ* must be less than 0.05 to make the proposed estimator efficient. Note that use of a small value of *ϖ* is possible if the majority of the NGO share auxiliary information because Eq (1) requires that (1−*ϖ*)% share data on auxiliary variable i.e., total revenue.

## IV. Numerical comparison of the proposed estimator with the estimator under imperfect ranked set sampling

For the purpose of numerically observing the behavior of relative efficiency, we are reporting the cases when *p*_*U*_ = *p*_*C*_ = *p*_*Com*_ = 0.5, so, the optimal weights are given as follows, *w*_1_ = *w*_2_ = *w*_3_ = 0.3333; where sum of weights is 1. Note that *p*_*U*_ is the probability that an NGO’s usability score exceeds the threshold set for the usability dimension of the index proposed by Nazuk and Shabbir [16] i.e., ${\hat{\mu }}_{NDI}$, similarly, *p*_*C*_ is the probability of exceeding the corresponding threshold for the dimension of content, while *P*_*Com*_ is the probability of exceeding the corresponding threshold for the dimension of communication. The reason we have analyzed the relative efficiency of the proposed index for *p*_*U*_ = *p*_*C*_ = *p*_*Com*_ = 0.5, is the fact that variance of ${\hat{\mu }}_{NDI}$ is an increasing function of *p*_*U*_, *p*_*C*_, and *P*_*Com*_, until 0.5, after which the variance starts decreasing. Therefore, we have considered the case when the variation in ${\hat{\mu }}_{NDI}$ is highest; this setting reflects the scenario when the NGO’ online disclosure practices are not strictly regulated, therefore, different NGO ${\hat{\mu }}_{NDI}$ scores vary significantly. For the countries where the nonprofit sector is highly regulated, values of *p*_*U*_, *p*_*C*_, and *P*_*Com*_>0.5 can be used to compute the optimal weights *w*_1_, *w*_2_, and *w*_3_.

In Table 2, one can observe that, other things remaining constant, as *ϖ* increases from 0.1 to 0.8, an approximate increase in the relative efficiency of ${\hat{\mu }}_{NGO.Disclosures}$ from 123 to 2500 takes place. When variation in online disclosures of NGO is higher, the improvement in relative efficiency is more significant. This means when the disclosure practices are not strictly regulated, then NGO follow organizational culture more than external pressures for sharing information, as a result there is significant variation in the online disclosure scores. Irrespective of the pattern in Table 2, it is readily evident that ${\hat{\mu }}_{NGO.Disclosures}$ performs much better than ${\hat{\mu }}_{IRSS}$; we have tested the response to very low values of *σ*_*Y*_, or (and) *σ*_*X*_, even in such scenarios ${\hat{\mu }}_{NGO.Disclosures}$ performs much better than ${\hat{\mu }}_{IRSS}$. In Table 3, we can observe that at *ϖ* = 0.8, an increase in $\rho_{XY}^{2}$ decreases the relative efficiency of the proposed estimator, nevertheless, the proposed estimator still remains efficient. A practical interpretation of Table 3 is that when $\rho_{XY}^{2}$ is high, then we must assign more weight to ${\hat{\mu }}_{IRSS}$ in Eq (1) because the auxiliary variable is strongly correlated with the study variable; therefore, it is logical to take maximum advantage of it. To better understand this, note the last row of Table 3, $\rho_{XY}^{2}$ is high but we keep on assigning more weight to ${\hat{\mu }}_{NDI}$ by using *ϖ* = 0.8, then we are not making the best use of the auxiliary variable, this has caused the relative efficiency to decrease as compared to the earlier rows in Table 3; despite this, the proposed estimator remains efficient than ${\hat{\mu }}_{IRSS}$.

**Table 2:** Relative efficiency of ${\hat{\mu }}_{NGO.Disclosures}$ in comparison to ${\hat{\mu }}_{IRSS}$; impact of increase in *σ*_*Y*_, and *ϖ*.

*σ*_*Y*_	*σ*_*X*_	$\rho_{XY}^{2}$	m	R	$\sum_{i=1}^{m}\sigma_{X\left(i:m\right)}^{2}$	*ϖ*	R.E
10	10	0.1	2	2	100	0.1	123.45
10	10	0.1	2	2	100	0.5	398.66
10	10	0.1	2	2	100	0.8	2372.84
100	10	0.1	2	2	100	0.1	123.46
100	10	0.1	2	2	100	0.5	399.99
100	10	0.1	2	2	100	0.8	2498.66
1000	10	0.1	2	2	100	0.1	123.46
1000	10	0.1	2	2	100	0.5	400.00
1000	10	0.1	2	2	100	0.8	2499.99
10000	10	0.1	2	2	100	0.1	123.46
10000	10	0.1	2	2	100.00	0.5	400.00
10000	10	0.1	2	2	100.00	0.8	2500.00

**Table 3:** Relative efficiency of ${\hat{\mu }}_{NGO.Disclosures}$ in comparison to ${\hat{\mu }}_{IRSS}$; impact of increase in $\rho_{XY}^{2}$ and *ϖ*.

*σ*_*Y*_	*σ*_*X*_	$\rho_{XY}^{2}$	m	r	$\sum_{i=1}^{m}\sigma_{X\left(i:m\right)}^{2}$	*ϖ*	R.E
10	10	0.1	2	2	100	0.8	2372.84
10	10	0.2	2	2	100	0.8	2370.99
10	10	0.3	2	2	100	0.8	2367.79
10	10	0.4	2	2	100	0.8	2363.04
10	10	0.5	2	2	100	0.8	2356.40
10	10	0.6	2	2	100	0.8	2347.36
10	10	0.7	2	2	100	0.8	2335.08
10	10	0.8	2	2	100	0.8	2318.21
10	10	0.9	2	2	100	0.8	2294.38

In Table 4, to analyze the performance of ${\hat{\mu }}_{NGO.Disclosures}$ with an increase in the value of $\rho_{XY}^{2}$, we simulated the values of relative efficiency for $\rho_{XY}^{2}$ between [0.1, 0.8]; for *ϖ* = 0.1, the value of relative efficiency is 123, approximately. For *ϖ* = 0.5, the value of relative efficiency is 400, approximately; while for *ϖ*>0.5, the value of relative efficiency is inversely proportional to $\rho_{XY}^{2}$, for instance, at *ϖ* = 0.8, as $\rho_{XY}^{2}$ increases the relative efficiency of ${\hat{\mu }}_{NGO.Disclosures}$ decreases, because if there is a higher correlation between online disclosures and total revenue we should weigh ${\hat{\mu }}_{IRSS}$ in Eq (1) but keeping *ϖ* = 0.8 means we are giving 80% weight to ${\hat{\mu }}_{NDI}.$ In Table 4, one can observe that when an increase in variation in total revenue i.e., *σ*_*X*_, is accompanied by an increase in *ϖ*, then the relative efficiency starts improving significantly. This means if there are NGO with diverse values of total revenue then we should rely more on ${\hat{\mu }}_{NDI}$; this means that in the presence of a heterogeneous sample, there is a need to introduce a stratified version of ${\hat{\mu }}_{NGO.Disclosures}$. However, one must observe that even the simple randomly sampled version of Eq (1), retains the efficiency of ${\hat{\mu }}_{NGO.Disclosures}$; in simple words even if one does not choose *ϖ* cautiously, performance of ${\hat{\mu }}_{NGO.Disclosures}$, remains better than ${\hat{\mu }}_{IRSS}$. In Tables 5 and 6, one can observe that an increase in *m* results in a loss in the relative efficiency of the proposed estimator; however, very large value of *m* is required to render the proposed estimator as inefficient.

**Table 4:** Relative efficiency of ${\hat{\mu }}_{NGO.Disclosures}$ in comparison to ${\hat{\mu }}_{IRSS}$; impact of increase in Var(X) and *ϖ*.

*σ*_*Y*_	*σ*_*X*_	$\rho_{XY}^{2}$	m	r	$\sum_{i=1}^{m}\sigma_{X\left(i:m\right)}^{2}$	*ϖ*	R.E
10	10	0.1	2	2	100	0.1	123.45
10	100	0.1	2	2	100	0.5	398.66
10	500	0.1	2	2	100	0.8	2372.23
10	1000	0.1	2	2	100	0.9	7857.47
**10**	**10**	0.5	**2**	**2**	**100**	0.1	123.45
10	100	0.5	2	2	100	0.5	398.23
10	500	0.5	2	2	100	0.8	2334.07
10	1000	0.5	2	2	100	0.9	7353.36
**10**	**10**	0.8	**2**	**2**	**100**	0.1	123.45
10	100	0.8	2	2	100	0.5	396.36
10	500	0.8	2	2	100	0.8	2177.57
10	1000	0.8	2	2	100	0.9	5714.98

**Table 5:** Relative efficiency of ${\hat{\mu }}_{NGO.Disclosures}$ in comparison to ${\hat{\mu }}_{IRSS}$; impact of increase in m.

*σ*_*Y*_	*σ*_*X*_	$\rho_{XY}^{2}$	m	r	$\sum_{i=1}^{m}\sigma_{X\left(i:m\right)}^{2}$	*ϖ*	R.E
10	100	0.1	2	5	1000	0.1	123.44
10	100	0.1	5	2	1000	0.1	123.44
10	100	0.1	10	2	1000	0.1	123.43
10	100	0.1	20	2	1000	0.1	123.41
10	100	0.1	50	2	1000	0.1	123.33
10	100	0.1	100	2	1000	0.1	123.20
10	100	0.1	150	2	1000	0.1	123.07
10	100	0.1	200	2	1000	0.1	122.95
10	100	0.1	300	2	1000	0.1	122.69
10	100	0.1	500	2	1000	0.1	122.19
10	100	0.1	1000	2	1000	0.1	120.94
10	100	0.1	11000	2	1000	0.1	100.49

**Table 6:** Relative efficiency of ${\hat{\mu }}_{NGO.Disclosures}$ in comparison to ${\hat{\mu }}_{IRSS}$; impact of increase in m, for larger variation in the study variable, and the auxiliary variable.

*σ*_*Y*_	*σ*_*X*_	$\rho_{XY}^{2}$	M	r	$\sum_{i=1}^{m}\sigma_{X\left(i:m\right)}^{2}$	*ϖ*	R.E
2000	1000	0.5	2	2	10000	0.1	123.46
2000	1000	0.5	5	2	10000	0.1	123.46
2000	1000	0.5	10	2	10000	0.1	123.46
2000	1000	0.5	20	2	10000	0.1	123.46
2000	1000	0.5	50	**2**	10000	0.1	123.46
2000	1000	0.5	100	2	10000	0.1	123.46
2000	1000	0.5	150	2	10000	0.1	123.46
2000	1000	0.5	200	2	10000	0.1	123.46
2000	1000	0.5	300	**2**	10000	0.1	123.46
2000	1000	0.5	500	2	10000	0.1	123.46
2000	1000	0.5	1000	2	10000	0.1	123.46
2000	1000	0.5	340000000	2	10000	0.1	100.11

## V. Guidelines to calculate the proposed index

To influence the policymakers and stakeholders, any statistical methodology must be easy to understand; therefore, we have summarized the proposed methodology in a concise manner. The following steps may be followed to adopt the proposed methodology.

1. Observe the percentage of NGO that: share auxiliary information on their websites versus those that do not share such information.
2. For those who do not share the auxiliary information, calculate NDI.
3. Those who share the information, randomly select *m* NGO, arrange these in ascending order of the auxiliary information, then choose the NGO with the minimum value of auxiliary information, and calculate it’s NDI.
4. Randomly select another set of "*m*" NGO, arrange these in ascending order of the auxiliary information, then choose the NGO with second minimum value of auxiliary information, and calculate its NDI. Similarly, select third minimum in the third set, fourth minimum in the fourth set, and so on up to the *m*^*th*^ set.
5. This completes one cycle of the imperfect ranked set sampling procedure.
6. Repeat step (3) to (4) "*r*" times to generate NDI values for "*mr*" NGO. Calculate the average score of disclosure scores through NDI in the set of "*mr*" NGO.
7. In the set of "*mr*" NGO, calculate: $\sigma_{Y}^{2}$ i.e., variance of online disclosure scores, $\rho_{XY}^{2}$ i.e., correlation between the online disclosure scores and the auxiliary information, $\sigma_{X}^{2}$ i.e., variance of auxiliary information, and $\sigma_{X\left(i:m\right)}^{2}$ i.e. variance of the *i*^*th*^ minimum value of the auxiliary variable, for instance, we can easily calculate the variance of *r* values corresponding to the 1^st^ minimum value of the auxiliary variable.
8. Use Eq (6) to calculate $V\left({\hat{\mu }}_{IRSS}\right)$.
9. Decide the thresholds for usability, content, and communication; if no such help is available through similar studies then use sample average, for instance, if the 32 indicators of content generate such values that their average is 2.5 then $p_{U}=\left[\begin{array}{c} 0,\;if\;NGO\;score\leq 2.5\\ 1,\;if\;NGO\;score>2.5 \end{array}\right]$. We can use such an average that is a fair representative of the balance point of data i.e., arithmetic mean if the data is not markedly skewed, or median in the case of markedly skewed distribution of NDI scores. Similarly, calculate *p*_*C*_, and *p*_*COM*_, then use Eq (3) to calculate optimum weights required for computation of NDI. Using the values of these probabilities, and weights, calculate variance of NDI through Eq (4).
10. Compare $V\left({\hat{\mu }}_{IRSS}\right)$ with $V\left({\hat{\mu }}_{NDI}\right)$.
11. Calculate $\frac{V\left({\hat{\mu }}_{NDI}\right)}{V\left({\hat{\mu }}_{IRSS}\right)}+1$, if it is less than 2, then we can safely use any value of *ϖ*. If $\frac{V\left({\hat{\mu }}_{NDI}\right)}{V\left({\hat{\mu }}_{IRSS}\right)}+1>2$, then look for a suitable value of *ϖ* required to render NDIA or ${\hat{\mu }}_{NGO.Disclosures}$, efficient, if there is a plausible value then compute NDIA or ${\hat{\mu }}_{NGO.Disclosures}$, otherwise rely only on NDI by taking *ϖ* = 1.

## VI. Discussion and conclusions

This paper discussed the potential of an index to monitor the online disclosure practices of non-governmental organizations (NGO), with a view to enable the stakeholders of the nonprofit sector to analyze different dimensions of accountability. Our approach offers a new methodology of monitoring accountability through the information shared on the NGO websites. Application of the proposed index i.e., NGO Disclosure Index under Auxiliary Information (NDIA), is possible with basic knowledge of mathematics; nevertheless, NGO (or the relevant regulatory body) can request support from a quantitative expert to calculate the value of NDIA. Significance of this study roots from the fact that there is qualified documented evidence of theoretical statisticians exploring the potential of complex sampling schemes to offer accountability related solutions to the nonprofit sector; moreover, a relatively simple guide for following the proposed methodology is showcased. Furthermore, technology can be used to facilitate application, e.g., an app can be built to help calculate scores. Similarly, automated measures for implementing our approach at a large (big data) scale can also be considered, e.g., AI (Artificial Intelligence) and NLP (Natural Language Processing) tools could be created to automatically analyze and score websites [45–47].

The post-Cold War era has witnessed a global inclination towards judicious use of authority by the governments. Several international and transnational regulatory organizations have emerged to control the global administrative space, such as the Basel Committee on Banking Supervision, The International Association of Insurance Fraud Agencies, United Nations, World Trade Organizations, The Financial Action Task Force on Money Laundering, Customs-Trade Partnership Against Terrorism, The Open Group, and World Customs Organization. Existence of so many regulatory organizations is sometimes debated as suboptimal, primarily because of the occasionally reported high profile negative events involving these organizations; nevertheless, one cannot label all these efforts as futile. Meaningful contributions from independent researchers are required to offer solutions for accountability paradigms that can facilitate the administration of the ambitious frameworks like GAL. In this context, the case of a global model of NGO regulation is even more intricate, because NGO regulatory frameworks are diverse even at a national level; a global model of NGO accountability requires an objective method through which stakeholders can evaluate the transparency of NGO. Although physical scrutiny (like audits and onsite inspections) of NGO across the world is a vital (albeit resource heavy) task; disclosure analysis of NGO websites can be a less demanding (yet highly effective) complementary pursuit. This study focuses on the importance of cyberspace; NGO disseminating information that may benefit various stakeholders in the hierarchy, for example, downward accountability towards the beneficiaries, and upward accountability towards regulatory bodies and relevant governmental departments. Although the proposed disclosure index is designed to analyze the quality of information shared online by the NGO, it can be used as a parallel form of accountability to conjoin or compare the information shared through different mediums i.e., online and offline. Moreover, it can help regulatory bodies create public information portals providing key information about NGO (including NDIA scores) in an accessible and transparent manner. National NDIA scores can be calculated for NGO working in different countries; the nonprofit sector in different countries can be ranked according to the quality of online disclosures. Similarly, a conglomeration of NGO can be defined according to different criteria, such as location of head office, thematic service areas, and years of work. NDIA can be calculated in each conglomerate, this can facilitate (even enable) informed policy actions, for instance, training the officials of a conglomerate with suboptimal performance about online disclosures.

The developed world has taken dedicated efforts to regulate the NGO sector through nonprofit accountability clubs and regulatory bodies, such as Global Reporting Initiatives, Charity Commission, GuideStar, Charity Review Council, GiveWell, Canada Revenue Agency, The Charity Commission of Northern Ireland, the Japan Association of Charitable Organizations, The Australian Charities and Not-for-Profits Commission, and Change Path. Although the developed world has taken considerable steps in the domain of the nonprofit sector’s regulation, episodes of suboptimal activities are still reported, such as the case of Greenpeace that faced public outrage due to the Brent Spar scandal in 1995 [48], or the case of a senior director in a Japanese NGO, who forged records and falsely declared stoneware, as priceless antiques [49]. In 2015, four charities that claimed to work for cancer patients faced charges of corruption; Federal Trade Commission of USA labeled Cancer Fund of America (CFA), Cancer Support Services (CSS), Children’s Cancer Fund of America (CCFOA), and The Breast Cancer Society (BCS) as duplicitous entities [50]. The developing and under-developed countries are increasingly becoming aware of the important task of NGO regulation; however, an arduous level of effort is required to regulate the nonprofit sector in such countries. For example, consider the case of Burundi, where NGO can only get registered by physically visiting Bujumbura (the capital city of Burundi), cost of registration is very high, and many documents required for registration are only available in hard copy from the Ministry of Home Affairs [51]. Even in some upper-middle-income countries [52], regulations of NGO require improvements, for instance, in the Republic of Columbia, the Public Registries of Chambers of Commerce is the core regulatory body responsible for registration of all types of nonprofit organizations; the exact number of NGO working in the country is not documented; moreover, the website of the Chambers of Commerce lacks a direct link to any nonprofit accountability club working in Columbia [53].

The vision of the world as a cosmopolitan community cannot be actualized without designing uniform standards of accountability; dedicated efforts are required for all sectors, including the global nonprofit sector. Attaining this level of efficient regulatory frameworks seems challenging, especially when the watchdogs are also doubted by the public; Blitt [54] presented the case for the need of efficient regulatory frameworks that can work without government intervention. This study is an effort to provide a statistically efficient, and pragmatic online solution to monitor the nonprofit sector; it showcases a low-cost solution for accountability, based on website analyses. Mathematical evaluation of the proposed index shows its ability to supersede simple estimators under imperfect ranked set sampling scheme. The idea purported by the current study relies on the segregation of indicators clubbed in the NDI, while the indicators are comprehensive; nevertheless, future researchers can add innovative details to improve the website analytics of NGO. Researchers can embed information technology with the proposed methodology, for instance, tools could be built for automated calculation of the online disclosure index scores. Similarly, AI and NLP tools could be created for proactive monitoring (and hence regulation) of NGO through semi or fully automated analysis of their website content [55, 56].

## Supporting information

S1 Data Simulations file plos one. (XLSX)
